# Pregnancy exposures and risk of childhood asthma admission in a population birth cohort

**DOI:** 10.1111/j.1399-3038.2011.01206.x

**Published:** 2011-12

**Authors:** Charles S Algert, Jennifer R Bowen, Samantha L Lain, Hugh D Allen, Josephine M Vivian-Taylor, Christine L Roberts

**Affiliations:** 1 Kolling Institute, University of SydneySydney, NSW, Australia; 2 Department of Obstetrics and Gynaecology, Royal North Shore HospitalSydney, NSW, Australia; 3 Department of Neonatology, Royal North Shore HospitalSydney, NSW, Australia; 4 Department of Paediatrics, Royal North Shore HospitalSydney, NSW, Australia

**Keywords:** asthma, children, population, pregnancy, risk factors

## Abstract

**Background:**

There is increasing interest in the potential for *in utero* exposures to affect the risk of asthma. We used population data to explore the associations between perinatal conditions and the risk of hospital admission with asthma between the 2nd and 5th birthday.

**Methods:**

The study population was 240,511 singleton infants born during 2001–2003. Birth records and longitudinally linked hospital admissions were used to identify asthma admissions and to model potential risk factors.

**Results:**

A total of 7245 children (3.0%) had one or more childhood admissions with asthma. *In utero* infectious exposures associated with childhood asthma were maternal antenatal admission with a urinary tract infection (UTI) [adjusted odds ratio (aOR) = 1.49, 95% confidence interval (1.23–1.79)] and pre-term pre-labor rupture of membranes (PROM) [aOR = 1.23 (1.04–1.45)]. There was no evidence that gestational age at time of first antenatal UTI admission (<28, ≥28 wks) affected the risk of asthma (homogeneity test p = 0.6). Pre-term birth was a risk factor for asthma admission, with the risk decreasing by 5.3% with each extra week of gestation. Autumn and winter conceptions were associated with an increased risk of childhood asthma admission: winter aOR = 1.15 (1.08–1.23), autumn aOR = 1.09 (1.02–1.16).

**Conclusions:**

As *in utero* exposure to both UTI and PROM carry an increased risk of childhood asthma admission, this suggests that the immune system response generally is the relevant factor rather than a specific organism. The season-associated risk is consistent with early pregnancy exposures such as the winter flu season or low vitamin D.

In the latter half of the 20th century, there has been such an increase in the prevalence of asthma in developed countries that it has been characterized as an ‘asthma epidemic,’ although part of the increase may be due to increased awareness of the symptoms of asthma and increased rates of reporting (1). There have been numerous studies investigating possible explanations for the increase but much remains unknown about the causes of asthma. Pregnancy is increasingly recognized as a time in which genes are activated or silenced. Such epigenetic programming can determine an individual's susceptibility to a wide range of clinical conditions. Study of early-life events is important both for an understanding of childhood asthma itself and of adult asthma (2). In addition to biologic factors such as parental history of asthma and male sex, childhood asthma is associated with environmental exposures and socio-economic factors. Potential risk factors specifically related to the delivery and neonatal period include prematurity, fetal growth restriction, and mode of delivery (3). Increased risk of childhood asthma may begin as early as an *in utero* exposure to maternal ascending infection, chorioamnionitis, and antibiotics that may lead to epigenetic changes in the fetus (4–7).

Large population-based studies using longitudinally linked population health databases enable the analysis of many potential perinatal risk factors for childhood asthma with sufficient numbers of events to minimize the chance of missing significant associations. The study of hospital admissions, representing inadequately controlled asthma and the severe end of the morbidity spectrum associated with asthma, particularly benefits from the availability of population data. Our aim was to develop a multivariable risk factor model of asthma hospitalization and to use the model to explore the strength of association with risk factors from the pregnancy and neonatal period.

## Methods

All singleton births to residents of New South Wales (NSW) Australia from 1 January 2001 through 31 December 2003 who survived to at least 2 yrs of age were included in the study population. Data were available from longitudinally linked population datasets: the Admitted Patient Data Collection, a database of all public and private hospital admissions in NSW, and the Midwives Data Collection that collects information on all births and is completed by the attending midwife or obstetrician. Information on infant cause of death was available through linkage to the Australian Bureau of Statistics (ABS) mortality data. As Australia does not have a unique registration number for citizens, the separate datasets were linked using probabilistic linkage methods. This involves a process of blocking and matching combinations of selected variables such as name, date of birth, address, and hospital and assigning a probability weight to the match. The validity of the probabilistic record linkage is extremely high with <1% of records having an incorrect match (8). The linked records were made available as de-identified data. Use of the data was approved by the NSW Population and Health Services Research Ethics Committee.

An admission was counted as an asthma-related admission if any of 20 diagnosis fields on a discharge record contained an International Classification of Diseases 10 (ICD-10) code for asthma (J45 or J46). Because of the uncertainty surrounding a diagnosis before the age of 24 months, hospital admissions for asthma before this age were not counted. Childhood hospitalizations were followed until the 5th birthday. All linked maternal discharge summaries (July 2000 to June 2009) were used to identify conditions specific to the pregnancy and also any maternal admission with an asthma code at any time during the study period.

### Potential perinatal risk factors

Maternal hypertension and maternal diabetes (including chronic and gestational for each condition) were identified from either the hospital discharge summaries or the birth record. Season of conception was calculated by subtracting the gestational age from the date of birth but adding 2 wks as an average time from last menstrual period to conception. Urinary tract infection (UTI) admissions were identified on the discharge summaries and included diagnoses of pyelonephritis and cystitis. Pre-labor rupture of membranes (PROM) was further categorized as term or pre-term (<37 wks gestation). Gestational age and small-for-gestational-age (SGA: <10th percentile birthweight for gestational age and sex) were included to represent the two underlying causes of low birthweight: prematurity and intrauterine growth retardation. Large-for-gestational-age (LGA: >90th percentile) was also included. Gestational age, mode of delivery, and labor induction were available from the birth record. Neonatal respiratory distress syndrome, transient tachypnea, sepsis, and jaundice were identified from the hospital discharge summaries. We used a combined exposure indicator for jaundice and/or phototherapy (9).

### Maternal and environmental factors of indeterminate timing

Environmental and some maternal socio-economic and health factors may affect pregnancy exposure but may also be proxies for childhood environmental exposures. For example, maternal smoking during the pregnancy has a bearing on whether the child is likely to grow up in a smoking household, and urban/rural differences could affect both pregnancy and childhood environments. Data on the birth record included smoking in pregnancy, parity, and maternal age. A socio-economic score was assigned to maternal postcode using the 2006 Socioeconomic Index for Areas (http://www.abs.gov.au) and then classified into quintiles. Residence at time of birth was categorized as urban or rural based upon the Accessibility/Remoteness Index of Australia score.

### Analysis

Crude relative risks (RR) and 95% confidence interval (95% CI) were calculated for each potential risk factor, for all births. Statistical evidence of a difference between two subgroup RR's was assessed using a chi-square test for homogeneity of effect. A hierarchical multilevel model was used to calculate adjusted odds ratios (aOR), simultaneously adjusting for all perinatal factors, using SAS's GLIMMIX procedure. A multilevel model takes into account that mothers within geographic areas may share environmental exposures and attend the same hospitals. The area-level grouping for this study was the ABS statistical local area. Season of conception and season of birth could not both be included in the regression model because of their direct correlation. Season of conception was selected for inclusion on the basis that this is the antecedent event. Gestational age in weeks was included as a continuous variable. Records with missing data (0.13%) were excluded from the regression model.

## Results

There were 244,878 eligible birth records but 4367 (1.8%), did not link to any hospital admission. This left 240,511 infants in the birth cohorts, among whom there were 7245 (3.0%) with at least one asthma admission between their 2nd and 5th birthday. The total number of admissions was 11,242; 5041 children had one admission, and 2204 had two or more admissions. The majority (69%) of the admissions were overnight or day only, but 1% of admissions were for 6 days or more and 126 (1.7%) children spent time in an intensive care unit. Most admissions (80%) were to a public hospital. There were two deaths in the birth cohort for whom asthma was listed as a contributing cause. Three infants died aged <1 yr with asthma listed as a contributing cause but were not included in the study cohorts because the deaths occurred before the minimum 2 yrs. There were 2963 children (1.2%) admitted with an asthma diagnosis at <2 yrs who then had no subsequent asthma admission and thus were not counted as cases in this analysis. Of the 7245 children admitted at ≥2 yrs, 1115 (27.3%) had also been admitted with an asthma diagnosis prior to their 2nd birthday.

Table 1 shows the crude RR for potential risk factors of an infant being admitted with asthma before 5 yrs of age. A history of any maternal admission with asthma doubled the risk of asthma in the child. Of the 217,387 mothers, 8326 (3.5%) had one or more admissions with asthma during the study period. The subgroup of 1453 women with two or more admissions had a risk of a child with an asthma admission of RR = 2.28 (1.87–2.77). There was a clear relationship between prematurity and risk of asthma admission, and for births ≤31 wks gestation, the risk was approximately triple. Even at term, there was an increased risk associated with birth before 40 wks gestation (RR = 1.11) that was important because 20% of births occurred at 37 through 38 wks gestation.

**Table 1:** Crude relative risks for admission to hospital with asthma between 2 and 5 yrs of age

Maternal/pregnancy/delivery factor	Infant asthma admissions/n	Crude relative risk RR (95% CI)
Maternal asthma admission*	508/8326	2.10 (1.93–2.30)
Maternal age (yrs)		
<20	381/10,210	1.25 (1.13–1.38)
20–34	5518/185,315	1.0 (referent)
≥35	1346/44,883	1.01 (0.95–1.07)
Parity		
Nulliparous (first child)	3018/100,115	1.01 (0.96–1.07)
Parity = 1	2473/81,462	1.02 (0.96–1.08)
Parity ≥ 2	1749/58,762	1.0 (referent)
Smoked during pregnancy	1308/38,468	1.16 (1.09–1.23)
Urban area resident	5146/164,667	1.13 (1.07–1.19)
Area socio-economic percentile		
>80th (most advantaged)	1360/49,008	0.87 (0.81–0.93)
>60th to 80th percentile	1360/47,297	0.90 (0.84–0.96)
40th to 60th percentile	1568/48,914	1.0 (referent)
20th to <40th percentile	1441/47,770	0.94 (0.88–1.01)
<20th (most disadvantaged)	1512/47,440	0.99 (0.93–1.07)
Any maternal diabetes	434/13,107	1.01 (1.00–1.22)
Any maternal hypertension	782/23,992	1.09 (1.02–1.17)
Season of conception		
Autumn	1796/58,445	1.09 (1.02–1.16)
Winter	1980/60,943	1.15 (1.08–1.23)
Spring	1745/59,908	1.03 (0.97–1.10)
Summer	1724/61,175	1.0 (referent)
Antenatal admit UTI†	123/2499	1.64 (1.38–1.96)
Rupture of membranes		
Pre-term PROM‡	189/3453	1.85 (1.61–2.13)
Term PROM‡	559/17,402	1.09 (1.00–1.18)
No PROM‡	6497/219,656	1.0 (referent)
Gestational age (wks)		
≤31	126/1459	3.11 (2.62–3.68)
32–33	88/1466	2.16 (1.76–2.65)
34–36	364/9303	1.41 (1.27–1.57)
37–39	3225/104,369	1.11 (1.06–1.17)
≥40	3442/123,875	1.0 (referent)
Induction with prostaglandins	812/27,708	0.97 (0.90–1.04)
Mode of delivery		
Cesarean section	1901/59,073	1.09 (1.04–1.15)
Instrumental	783/25,864	1.03 (0.96–1.11)
Normal vaginal	4560/155,533	1.0 (referent)
Male infant	4661/124,114	1.69 (1.61–1.77)
Birthweight percentile		
SGA (<10th percentile)	805/24,118	1.12 (1.04–1.20)
10th–90th	5721/191,604	1.0 (referent)
LGA (>90th percentile)	719/24,746	0.97 (0.90–1.05)
Respiratory distress syndrome	193/3062	2.12 (1.85–2.44)
Transient tachypnea	306/7271	1.41 (1.26–1.58)
Jaundice and/or phototherapy	548/12,831	1.45 (1.33–1.58)
Neonatal bacterial sepsis	57/957	1.99 (1.54–2.56)

LGA, large-for-gestational-age’ PROM, pre-labor rupture of membranes; SGA, small-for-gestational-age.

* Mother was admitted with asthma anytime between July 2000 and June 2009.

† Urinary tract infection.

‡ Spontaneous pre-labor rupture of membranes.

We analyzed the risk of ever being admitted for asthma in relation to season and found evidence of seasonal variation. By estimated month of conception, with summer as the referent season, the risk of childhood asthma admission for conceptions in other seasons was as follows: spring RR = 1.03 (0.97–1.10), autumn RR = 1.09 (1.02–1.16), and winter RR = 1.15 (1.08–1.23). By month of birth, with winter as the referent season, the risk for other birth seasons was as follows: spring RR = 0.99 (0.93–1.06), summer RR = 1.09 (1.02–1.17), and autumn RR = 1.13 (1.06–1.20).

Women with any antenatal UTI admission(s) were further categorized into subgroups by whether the first admission was before 28 wks (first two trimesters of pregnancy) or not. Women with a UTI before 28 wks gestation had RR = 1.73 (1.33–2.33) of a child with an asthma admission compared with women with no UTI admission, and women with a first UTI admission at ≥28 wks had RR = 1.58 (1.26–2.00); there was no evidence of a difference (homogeneity chi square p = 0.6). We also explored a potential dose–response to antenatal UTI. Women with two or more antenatal UTI admissions in the same pregnancy had children with RR = 2.51 (1.66–3.78) of later asthma admission compared with women without a UTI admission; women with only one antenatal UTI admission had RR = 1.54 (1.27–1.86). The test of homogeneity showed borderline evidence (p = 0.04) of a difference in effect.

Table 2 shows the adjusted risks from the logistic regression model of childhood asthma admission. For parity, any women with a previous birth were combined into one referent category, resulting in a non-significant RR = 0.97 (0.92–1.02) for first-born infants. For the socio-economic indicator, the lower three quintiles were combined into one referent category. The two most affluent socio-economic quintiles remained protective after adjustment for other factors. Gestational age was included as a continuous variable; the aOR = 0.947 means that the risk decreased by 5.3% (95% CI 3.8–6.7%) for each extra week of gestation. The crude risk ratio for most factors changed by ≤10% after adjustment in the model. The two strongest risk factors, maternal asthma admission and male child, had adjusted risks that were almost unchanged from their crude RR's. Factors for which the adjusted risk decreased by >10% included pre-term PROM, transient tachypnea, respiratory distress syndrome, neonatal sepsis, and jaundice/phototherapy. The risk for neonatal sepsis dropped from the crude RR = 1.99 to an aOR = 1.20 with a confidence interval that included unity.

**Table 2:** Adjusted odds ratios (aOR) for admission to hospital with asthma between 2 and 5 yrs of age (simultaneous adjustment for all listed factors in the entire table)

	aOR of asthma admission (95% CI)
Maternal and environmental factors	
Maternal asthma admission*	2.09 (1.90–2.29)
Maternal age <20 yrs	1.20 (1.07–1.34)
Maternal age ≥35 yrs	1.00 (0.94–1.07)
Nulliparous (first child)	0.97 (0.92–1.02)
Smoked during pregnancy	1.08 (1.02–1.15)
Urban area resident	1.18 (1.10–1.26)
Most advantaged socio-economic quintile	0.87 (0.80–0.95)
2nd advantaged socio-economic quintile	0.92 (0.86–0.99)
Pregnancy and delivery factors	
Spring conception	1.03 (0.96–1.10)
Autumn conception	1.09 (1.02–1.16)
Winter conception	1.15 (1.08–1.23)
Antenatal admit UTI†	1.49 (1.23–1.79)
Any maternal diabetes	0.95 (0.86–1.06)
Any maternal hypertension	1.02 (0.95–1.11)
Pre-term PROM‡	1.23 (1.04–1.45)
Term PROM‡	1.06 (0.97–1.16)
Induction with prostaglandins	1.05 (0.97–1.13)
Cesarean section delivery	1.03 (0.97–1.09)
Infant factors	
Male	1.70 (1.62–1.79)
Gestational age (per additional week)	0.947 (0.93–0.96)
SGA (<10th percentile size)	1.08 (1.00–1.17)
LGA (>10th percentile size)	0.97 (0.89–1.05)
Respiratory distress syndrome	1.34 (1.12–1.60)
Transient tachypnea of newborn	1.20 (1.07–1.36)
Jaundice and/or phototherapy	1.10 (0.99–1.22)
Neonatal bacterial sepsis	1.20 (0.90–1.60)

LGA, large-for-gestational-age; PROM, pre-labor rupture of membranes; SGA, small-for-gestational-age.

* Mother was admitted with asthma anytime between July 2000 and June 2009.

† Urinary tract infection

‡ Spontaneous pre-labor rupture of membranes.

## Discussion

In our large population-based study, we find that an increased risk of asthma admission in children is associated with birth factors including *in utero* exposures, even after adjustment for other maternal and pregnancy factors. Among the factors related to birth, the largest increases in risk are associated with pre-term birth and with fetal exposure to maternal UTI and PROM. Previous findings on pre-term birth have been mixed but a meta-analysis did find an overall association with childhood asthma (10). Both pre-term birth and rupture of membranes may be triggered by intrauterine infection and resultant chorioamnionitis (11), so that all these factors are inter-related. Intrauterine infections have separately been reported to be associated with increased risk of childhood asthma (4–6). That both maternal UTI and PROM are associated with an increased risk of childhood asthma in our population suggests that the risk associated with maternal infection is not organism-specific but may be related to the inflammatory response generally. The most common microbe responsible for UTI is *Escherichia coli*, an organism not commonly found as an intrauterine pathogen (4).

The antenatal period is a critical window for programming of epigenetic changes (12), and both maternal UTI and any infection associated with PROM may have multiple effects on the fetus related to *in utero* infection, bacterial colonization, and exposure to inflammatory cytokines. The timing of UTI exposure in pregnancy did not appear to be critical in our study population as the point estimate for first UTI admission at <28 wks is not significantly different to that for first UTI at ≥28 wks. There is borderline evidence of a dose–response association, as we found that women with two or more antenatal UTI admissions have a greater risk of asthma admission for their child. An apparent dose–response relation has been reported between *in utero* exposure to antibiotic treatment and subsequent asthma (6), and maternal UTI treated with antibiotics has also been associated with transient early wheezing (7). The point estimate for the adjusted risk associated with pre-term PROM in our study is more than triple that for term PROM (aOR = 1.23 vs. aOR = 1.06), suggesting that timing or duration of exposure to ruptured membranes may contribute to an increased risk of asthma. A recent study has reported that duration of ruptured membranes before birth is positively associated with the prevalence of wheezing at 18 months of age (13). During the study period, women with pre-term PROM usually received antibiotics (14) including multiple courses if delivery did not occur until weeks after PROM, although this does not ensure eradication or prevention of intra-amniotic infection (15). Importantly, follow-up of the ORACLE I placebo-controlled trial (antibiotics for pre-term PROM) found no difference in parent-reported wheezing at 7 yrs of age by antibiotic treatment exposure (16). Follow-up of the ORACLE II trial (antibiotics for pre-term labor with intact membranes) similarly found no increased risk by antibiotic exposure (17). This supports the hypothesis that it is infection rather than the antibiotics prescribed for treatment that is responsible for the asthma risk.

There is a seasonal association with the risk of childhood asthma in our birth cohorts. The association exists both by month of conception and by month of delivery. By season of conception, the highest seasonal risk was for winter (aOR = 1.15), and by season of birth, the highest risk was autumn (aOR = 1.13). One possible explanation for variation by season of conception may be reduced sun exposure during the first trimester of pregnancy resulting in lower levels of vitamin D. *In utero* vitamin D deficiency has been hypothesized to be a risk factor for asthma (18). Another possible explanation is that women who conceive in autumn or winter are exposed to the peak flu season in their first trimester. Maternal infectious disease in pregnancy, including febrile disease, has been associated with increased risk of asthma at age 7 (19), although another study found no association between self-reported febrile disease in the 2nd and 3rd trimester of pregnancy and wheeze in the child during the first 2 yrs (20). By month of birth, the peak increase in asthma risk is for autumn (RR = 1.13), and the direction of this effect is consistent with a report that birth 4 months before the winter virus peak season had aOR = 1.29 (1.19–1.40) of childhood hospitalization with asthma (21) Conception and delivery are inextricably linked; a birth 4 months before mid-winter implies a winter conception. Our data do not permit us to determine which of the exposure windows is the more relevant. However, reports that cytokine responses and Immunoglobulin E levels from neonatal cord blood showed seasonal variation suggests that at least some of the seasonal exposure occurs *in utero* (22, 23).

Respiratory distress syndrome and transient tachypnea are both independent neonatal risk factors for later asthma hospitalization in our study although the strength of association did moderate after adjustment for other factors such as gestational age. Transient tachypnea, which is nominally a temporary condition of the newborn, carries an aOR = 1.20 of asthma admission. The direction of this effect is consistent with the aOR = 1.50 (1.13–1.99) reported by a nested case–control study.

An interesting finding in our study is that being a first-born child (a nulliparous mother) does not result in an increased risk of childhood hospitalization for asthma. This appears opposed to the protective effect of older siblings posited by the hygiene hypothesis but may simply reflect that hospitalization with asthma is not the same outcome as diagnosis by primary care providers. Colds, flu, and sinus infections are predictors of uncontrolled asthma (24), and it may be that any protective effect that could be attributed to the hygiene hypothesis is more than offset by an increased risk of an RTI when there are siblings. Higher socio-economic status is associated with reduced asthma hospitalization in our study population, and this may be related to better control of asthma as much as to variations in prevalence. A UK study found a similar protective effect for children (aged ≥2 yrs) of families in the top two social classes: aOR = 0.81 (0.74–0.89) of hospital admission with asthma (25).

A strength of our study is that it is population-based and has a clearly defined dichotomous outcome (hospital admission) and that validated exposure factors (26, 27) were determined at the time of pregnancy and delivery and so were unbiased by subsequent asthma status in the children. A limitation to its generalizability is that the outcome represents children with inadequately controlled and severe episodes of asthma, rather than all children with asthma. Asthma phenotypes could not be characterized, as we did not have information on the medications used for control. Hospital admissions can include a heterogenous mixture of children with chronic asthma and those with infrequent acute episodes (28). Our study population is recent, and so the findings are relevant to current obstetric practices, but follow-up does not extend beyond the 5th birthday. A further limitation is that maternal medical data outside of hospital was not available. Infections and antibiotic treatment are not uncommon in pregnancy but do not generally result in hospital admission. In an English pregnancy cohort, 42.2% of women reported an infection and 35.1% reported use of antibiotics (6). The theoretically unexposed comparison groups for UTI and PROM exposures in our study probably included women who had less severe UTI's treated out of hospital, or who had subclinical chorioamnionitis, or who had received antibiotics for other infections. The likely, but not certain, result of such misclassified exposure would be that our reported risks underestimate the true risks for potential fetal infectious exposures. Maternal asthma status could not be fully ascertained from our data. An Australian single-hospital study found that in a sample of women attending for antenatal care, 12.7% reported asthma and 7.8% used airway medications (29) in comparison with the 3.5% of mothers in our study identified as having a history of admission with asthma. It is likely that fewer than half of women on asthma medication would have been identified based upon admission history, and that the women with admissions may introduce some selection bias into the adjusted model of asthma risk. A further limitation is that other factors of interest such as breastfeeding and paternal asthma were not available in our datasets.

In summary, our large population-based study strengthens the case that birth exposures are associated with childhood asthma and suggests that increased risk may begin with fetal exposures to a range of maternal infections. An intriguing question is whether the seasonal influence on risk of developing childhood asthma could be related to either early pregnancy exposure to the winter flu season or to low winter levels of vitamin D.
